# 

**Published:** 1874-06

**Authors:** 


### NORTHERN OHIO DENTAL ASSOCIATION.

The fifteenth annual meeting of the Northern Ohio Dental Association will be held at Put-in Bay Island, commencing on Tuesday, June 9th, 1874, at 10 o'clock, A. M.

Session will continue two days, and a full attendance is desired.

Members of the profession are cordially invited to be present. S. P. Hildreth, President; C. Buffett, Corresponding Secretary.

ORDER OF BUSINESS.

Reading of Minutes of last Annual Meeting.

Reports of Officers and Committees.

Reading of Essays and Discussions.

Miscellaneous Business.

Election of Officers 11 A. M., Second day.

SUBJECTS FOR DISCUSSION.

The Care of Teeth during the period of Dental Development.

Separating Teeth preparatory to Filling.

Can Fillings perfectly impervious to moisture, be made with Cohesive Gold?

Dental Ethics.

Miscellaneous.

